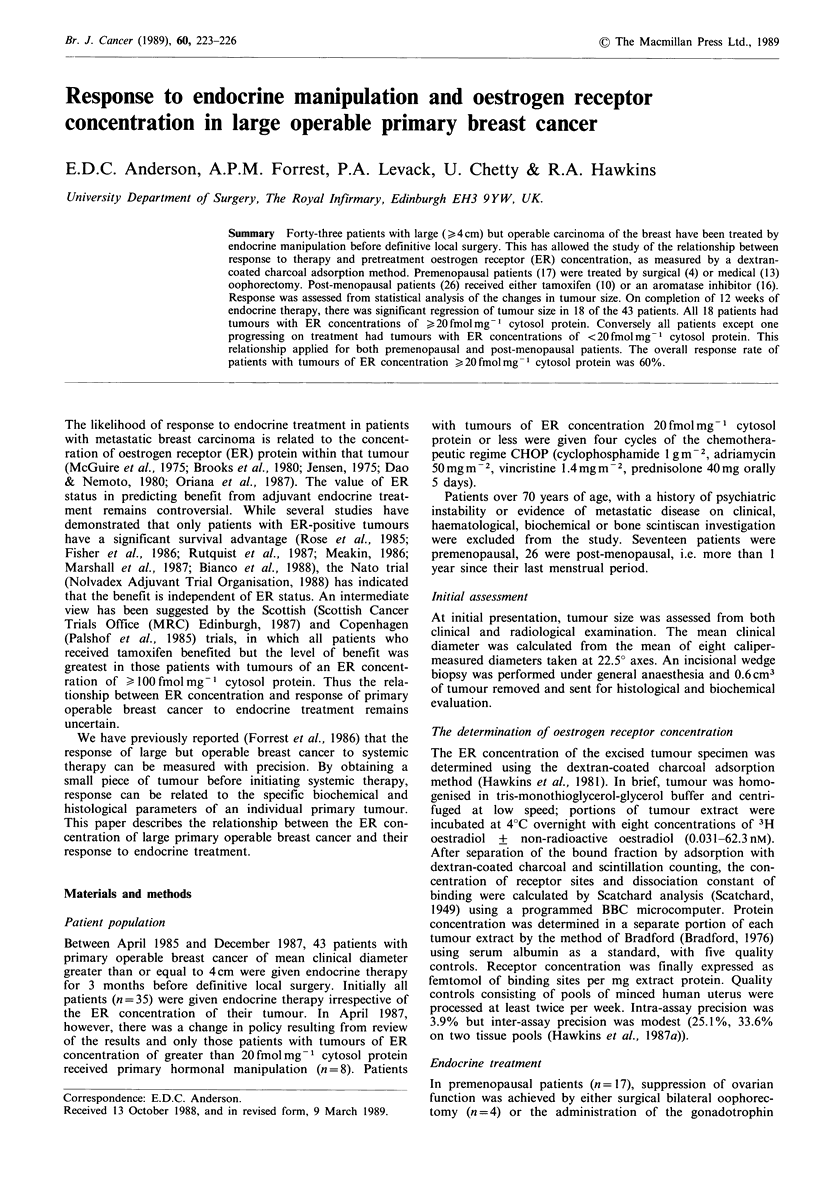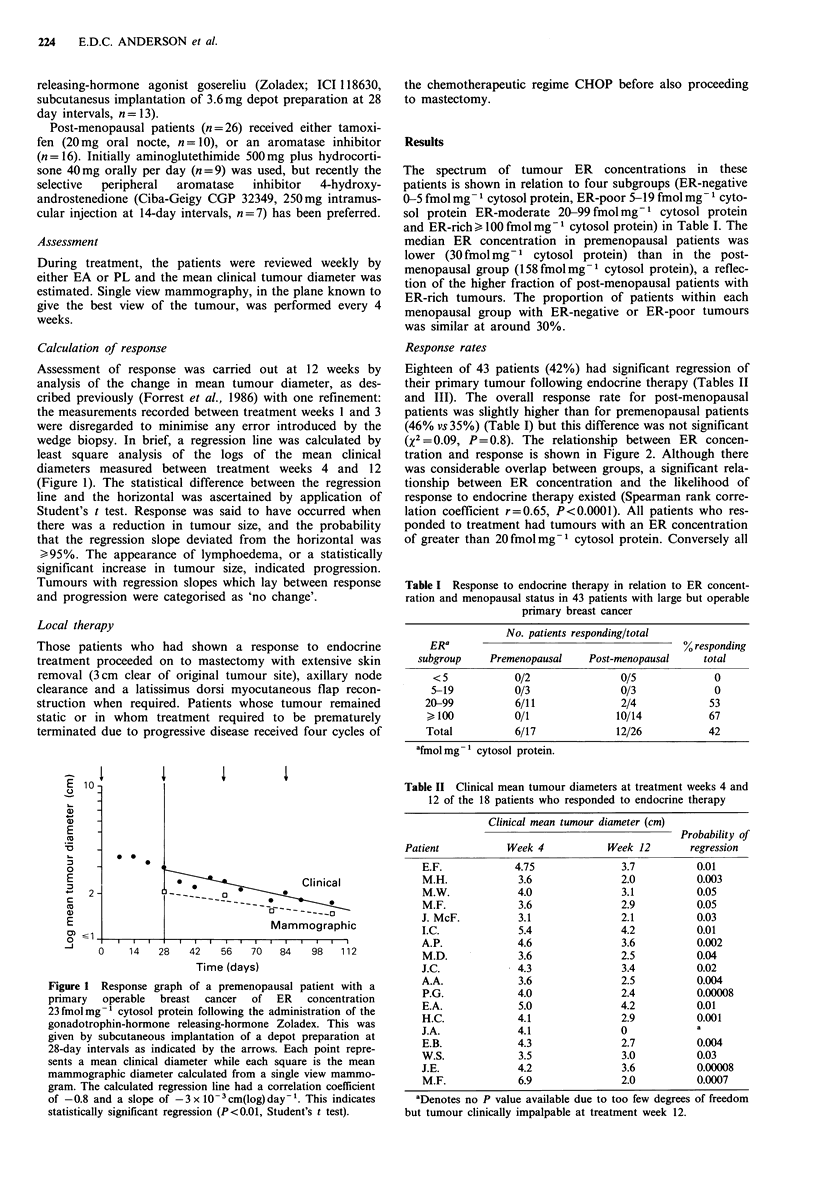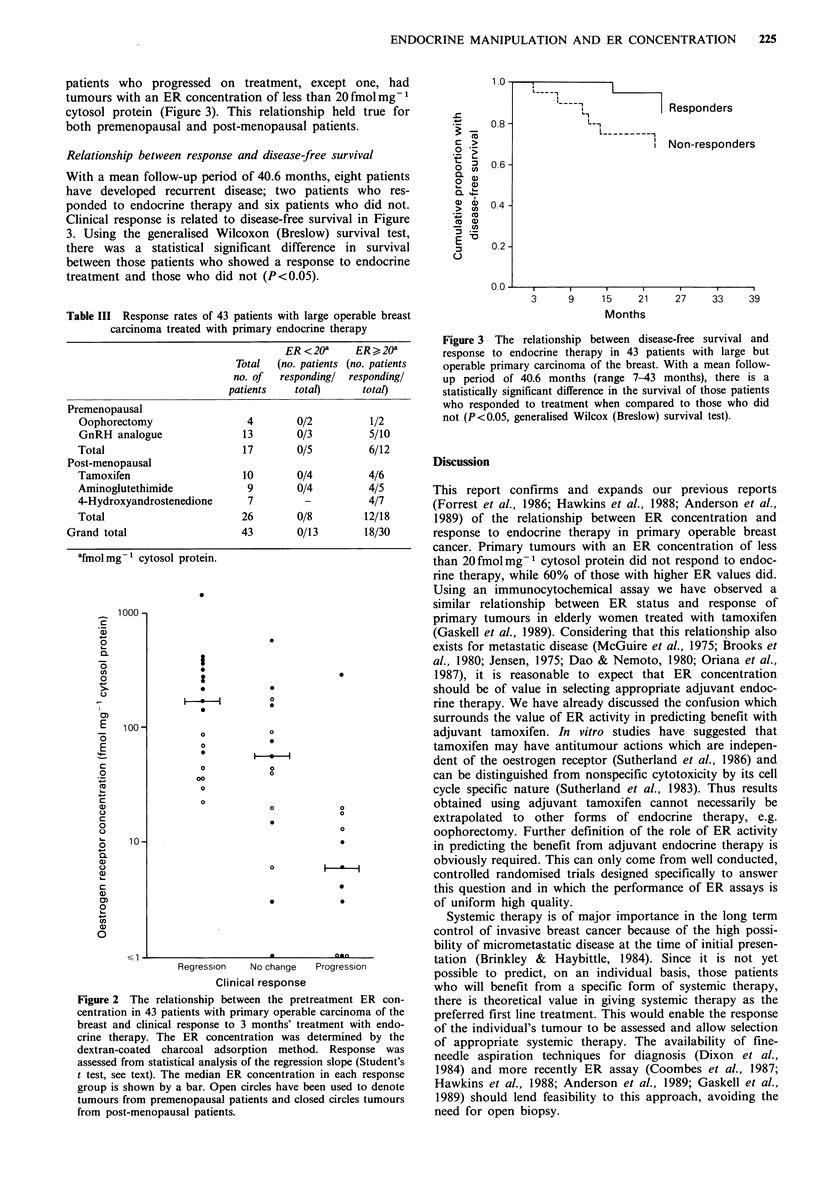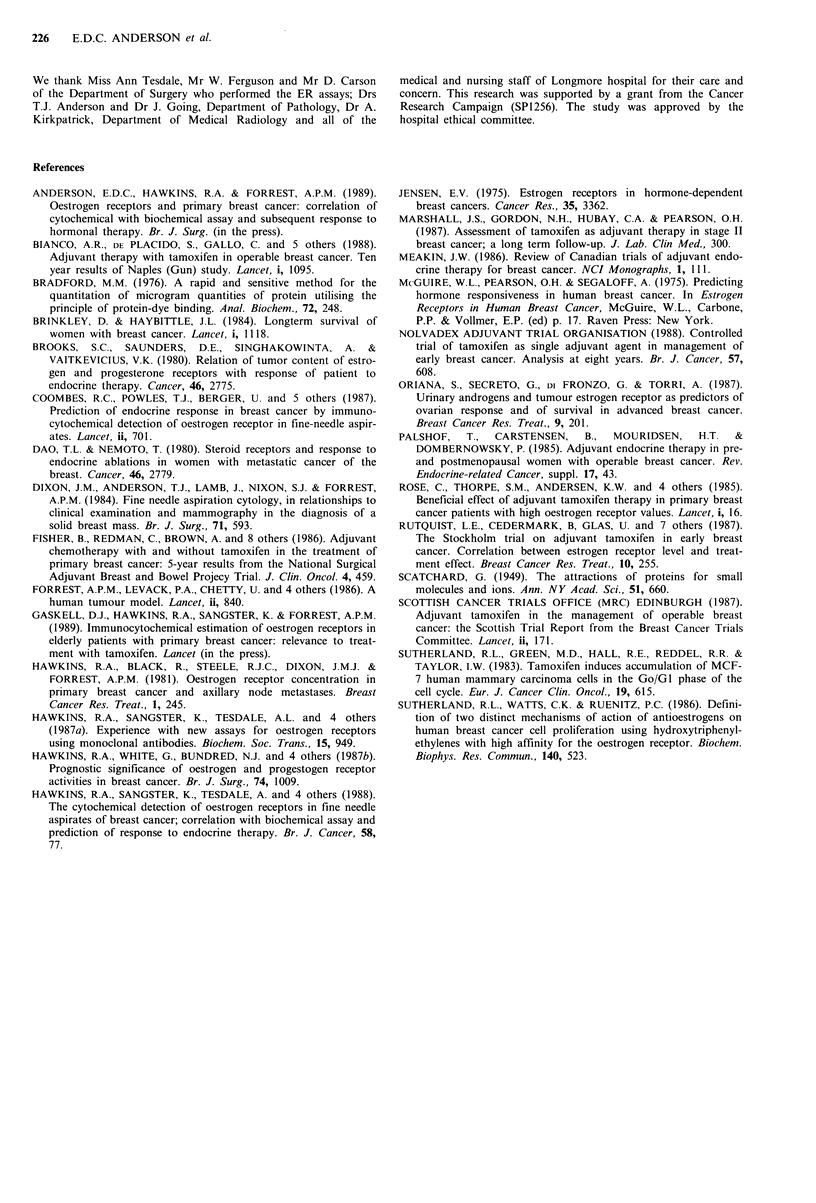# Response to endocrine manipulation and oestrogen receptor concentration in large operable primary breast cancer.

**DOI:** 10.1038/bjc.1989.256

**Published:** 1989-08

**Authors:** E. D. Anderson, A. P. Forrest, P. A. Levack, U. Chetty, R. A. Hawkins

**Affiliations:** University Department of Surgery, Royal Infirmary, Edinburgh, UK.

## Abstract

Forty-three patients with large (greater than or equal to 4 cm) but operable carcinoma of the breast have been treated by endocrine manipulation before definitive local surgery. This has allowed the study of the relationship between response to therapy and pretreatment oestrogen receptor (ER) concentration, as measured by a dextran-coated charcoal adsorption method. Premenopausal patients (17) were treated by surgical (4) or medical (13) oophorectomy. Post-menopausal patients (26) received either tamoxifen (10) or an aromatase inhibitor (16). Response was assessed from statistical analysis of the changes in tumour size. On completion of 12 weeks of endocrine therapy, there was significant regression of tumour size in 18 of the 43 patients. All 18 patients had tumours with ER concentrations of greater than or equal to 20 fmol mg-1 cytosol protein. Conversely all patients except one progressing on treatment had tumours with ER concentrations of less than 20 fmol mg-1 cytosol protein. This relationship applied for both premenopausal and post-menopausal patients. The overall response rate of patients with tumours of ER concentration greater than or equal to 20 fmol mg-1 cytosol protein was 60%.


Br. J. Cancer (1989), 60, 223–226 © The Macmillan Press Ltd., 1989

### Response to endocrine manipulation and oestrogen receptor concentration in large operable primary breast cancer

E.D.C. Anderson, A.P.M. Forrest, P.A. Levack, U. Chetty & R.A. Hawkins

*University Department of Surgery, The Royal Infirmary, Edinburgh EH3 9YW, UK.*

**Summary** Forty-three patients with large (≥4 cm) but operable carcinoma of the breast have been treated by endocrine manipulation before definitive local surgery. This has allowed the study of the relationship between response to therapy and pretreatment oestrogen receptor (ER) concentration, as measured by a dextran-coated charcoal adsorption method. Premenopausal patients (17) were treated by surgical (4) or medical (13) oophorectomy. Post-menopausal patients (26) received either tamoxifen (10) or an aromatase inhibitor (16). Response was assessed from statistical analysis of the changes in tumour size. On completion of 12 weeks of endocrine therapy, there was significant regression of tumour size in 18 of the 43 patients. All 18 patients had tumours with ER concentrations of $\geqslant 20\,\text{fmol}\,\text{mg}^{-1}$ cytosol protein. Conversely all patients except one progressing on treatment had tumours with ER concentrations of $<20\,\text{fmol}\,\text{mg}^{-1}$ cytosol protein. This relationship applied for both premenopausal and post-menopausal patients. The overall response rate of patients with tumours of ER concentration $\geqslant 20\,\text{fmol}\,\text{mg}^{-1}$ cytosol protein was 60%.

The likelihood of response to endocrine treatment in patients with metastatic breast carcinoma is related to the concentration of oestrogen receptor (ER) protein within that tumour (McGuire *et al.*, 1975; Brooks *et al.*, 1980; Jensen, 1975; Dao & Nemoto, 1980; Oriana *et al.*, 1987). The value of ER status in predicting benefit from adjuvant endocrine treatment remains controversial. While several studies have demonstrated that only patients with ER-positive tumours have a significant survival advantage (Rose *et al.*, 1985; Fisher *et al.*, 1986; Rutquist *et al.*, 1987; Meakin, 1986; Marshall *et al.*, 1987; Bianco *et al.*, 1988), the Nato trial (Nolvadex Adjuvant Trial Organisation, 1988) has indicated that the benefit is independent of ER status. An intermediate view has been suggested by the Scottish (Scottish Cancer Trials Office (MRC) Edinburgh, 1987) and Copenhagen (Palshof *et al.*, 1985) trials, in which all patients who received tamoxifen benefited but the level of benefit was greatest in those patients with tumours of an ER concentration of $\geqslant 100\,\text{fmol}\,\text{mg}^{-1}$ cytosol protein. Thus the relationship between ER concentration and response of primary operable breast cancer to endocrine treatment remains uncertain.

We have previously reported (Forrest *et al.*, 1986) that the response of large but operable breast cancer to systemic therapy can be measured with precision. By obtaining a small piece of tumour before initiating systemic therapy, response can be related to the specific biochemical and histological parameters of an individual primary tumour. This paper describes the relationship between the ER concentration of large primary operable breast cancer and their response to endocrine treatment.

### Materials and methods

### *Patient population*

Between April 1985 and December 1987, 43 patients with primary operable breast cancer of mean clinical diameter greater than or equal to 4 cm were given endocrine therapy for 3 months before definitive local surgery. Initially all patients ($n=35$) were given endocrine therapy irrespective of the ER concentration of their tumour. In April 1987, however, there was a change in policy resulting from review of the results and only those patients with tumours of ER concentration of greater than $20\,\text{fmol}\,\text{mg}^{-1}$ cytosol protein received primary hormonal manipulation ($n=8$). Patients with tumours of ER concentration $20\,\text{fmol}\,\text{mg}^{-1}$ cytosol protein or less were given four cycles of the chemotherapeutic regime CHOP (cyclophosphamide $1\,\text{g}\,\text{m}^{-2}$, adriamycin $50\,\text{mg}\,\text{m}^{-2}$, vincristine $1.4\,\text{mg}\,\text{m}^{-2}$, prednisolone 40 mg orally 5 days).

Patients over 70 years of age, with a history of psychiatric instability or evidence of metastatic disease on clinical, haematological, biochemical or bone scintiscan investigation were excluded from the study. Seventeen patients were premenopausal, 26 were post-menopausal, i.e. more than 1 year since their last menstrual period.

### *Initial assessment*

At initial presentation, tumour size was assessed from both clinical and radiological examination. The mean clinical diameter was calculated from the mean of eight caliper-measured diameters taken at 22.5° axes. An incisional wedge biopsy was performed under general anaesthesia and $0.6\,\text{cm}^3$ of tumour removed and sent for histological and biochemical evaluation.

### *The determination of oestrogen receptor concentration*

The ER concentration of the excised tumour specimen was determined using the dextran-coated charcoal adsorption method (Hawkins *et al.*, 1981). In brief, tumour was homogenised in tris-monothioglycerol-glycerol buffer and centrifuged at low speed; portions of tumour extract were incubated at 4°C overnight with eight concentrations of $^3$H oestradiol ± non-radioactive oestradiol (0.031–62.3 nM). After separation of the bound fraction by adsorption with dextran-coated charcoal and scintillation counting, the concentration of receptor sites and dissociation constant of binding were calculated by Scatchard analysis (Scatchard, 1949) using a programmed BBC microcomputer. Protein concentration was determined in a separate portion of each tumour extract by the method of Bradford (Bradford, 1976) using serum albumin as a standard, with five quality controls. Receptor concentration was finally expressed as femtomol of binding sites per mg extract protein. Quality controls consisting of pools of minced human uterus were processed at least twice per week. Intra-assay precision was 3.9% but inter-assay precision was modest (25.1%, 33.6% on two tissue pools (Hawkins *et al.*, 1987*a*)).

### *Endocrine treatment*

In premenopausal patients ($n=17$), suppression of ovarian function was achieved by either surgical bilateral oophorectomy ($n=4$) or the administration of the gonadotrophin

Correspondence: E.D.C. Anderson.
Received 13 October 1988, and in revised form, 9 March 1989.

releasing-hormone agonist gosereliu (Zoladex; ICI 118630, subcutanesus implantation of 3.6 mg depot preparation at 28 day intervals, $n = 13$).

Post-menopausal patients ($n = 26$) received either tamoxifen (20 mg oral nocte, $n = 10$), or an aromatase inhibitor ($n = 16$). Initially aminoglutethimide 500 mg plus hydrocortisone 40 mg orally per day ($n = 9$) was used, but recently the selective peripheral aromatase inhibitor 4-hydroxy-androstenedione (Ciba-Geigy CGP 32349, 250 mg intramuscular injection at 14-day intervals, $n = 7$) has been preferred.

*Assessment*

During treatment, the patients were reviewed weekly by either EA or PL and the mean clinical tumour diameter was estimated. Single view mammography, in the plane known to give the best view of the tumour, was performed every 4 weeks.

*Calculation of response*

Assessment of response was carried out at 12 weeks by analysis of the change in mean tumour diameter, as described previously (Forrest *et al.*, 1986) with one refinement: the measurements recorded between treatment weeks 1 and 3 were disregarded to minimise any error introduced by the wedge biopsy. In brief, a regression line was calculated by least square analysis of the logs of the mean clinical diameters measured between treatment weeks 4 and 12 (Figure 1). The statistical difference between the regression line and the horizontal was ascertained by application of Student's $t$ test. Response was said to have occurred when there was a reduction in tumour size, and the probability that the regression slope deviated from the horizontal was ≥95%. The appearance of lymphoedema, or a statistically significant increase in tumour size, indicated progression. Tumours with regression slopes which lay between response and progression were categorised as 'no change'.

*Local therapy*

Those patients who had shown a response to endocrine treatment proceeded on to mastectomy with extensive skin removal (3 cm clear of original tumour site), axillary node clearance and a latissimus dorsi myocutaneous flap reconstruction when required. Patients whose tumour remained static or in whom treatment required to be prematurely terminated due to progressive disease received four cycles of the chemotherapeutic regime CHOP before also proceeding to mastectomy.

**Figure 1** Response graph of a premenopausal patient with a primary operable breast cancer of ER concentration 23 fmol mg$^{-1}$ cytosol protein following the administration of the gonadotrophin-hormone releasing-hormone Zoladex. This was given by subcutaneous implantation of a depot preparation at 28-day intervals as indicated by the arrows. Each point represents a mean clinical diameter while each square is the mean mammographic diameter calculated from a single view mammogram. The calculated regression line had a correlation coefficient of $-0.8$ and a slope of $-3 \times 10^{-3}$ cm(log) day$^{-1}$. This indicates statistically significant regression ($P < 0.01$, Student's $t$ test).

### Results

The spectrum of tumour ER concentrations in these patients is shown in relation to four subgroups (ER-negative 0–5 fmol mg$^{-1}$ cytosol protein, ER-poor 5–19 fmol mg$^{-1}$ cytosol protein ER-moderate 20–99 fmol mg$^{-1}$ cytosol protein and ER-rich ≥ 100 fmol mg$^{-1}$ cytosol protein) in Table I. The median ER concentration in premenopausal patients was lower (30 fmol mg$^{-1}$ cytosol protein) than in the post-menopausal group (158 fmol mg$^{-1}$ cytosol protein), a reflection of the higher fraction of post-menopausal patients with ER-rich tumours. The proportion of patients within each menopausal group with ER-negative or ER-poor tumours was similar at around 30%.

*Response rates*

Eighteen of 43 patients (42%) had significant regression of their primary tumour following endocrine therapy (Tables II and III). The overall response rate for post-menopausal patients was slightly higher than for premenopausal patients (46% *vs* 35%) (Table I) but this difference was not significant ($\chi^2 = 0.09$, $P = 0.8$). The relationship between ER concentration and response is shown in Figure 2. Although there was considerable overlap between groups, a significant relationship between ER concentration and the likelihood of response to endocrine therapy existed (Spearman rank correlation coefficient $r = 0.65$, $P < 0.0001$). All patients who responded to treatment had tumours with an ER concentration of greater than 20 fmol mg$^{-1}$ cytosol protein. Conversely all

**Table I** Response to endocrine therapy in relation to ER concentration and menopausal status in 43 patients with large but operable primary breast cancer

| *ER[a] subgroup* | *No. patients responding/total* | | *% responding total* |
|---|---|---|---|
| | *Premenopausal* | *Post-menopausal* | |
| <5 | 0/2 | 0/5 | 0 |
| 5–19 | 0/3 | 0/3 | 0 |
| 20–99 | 6/11 | 2/4 | 53 |
| ≥100 | 0/1 | 10/14 | 67 |
| Total | 6/17 | 12/26 | 42 |

[a]fmol mg$^{-1}$ cytosol protein.

**Table II** Clinical mean tumour diameters at treatment weeks 4 and 12 of the 18 patients who responded to endocrine therapy

| *Patient* | *Clinical mean tumour diameter (cm)* | | *Probability of regression* |
|---|---|---|---|
| | *Week 4* | *Week 12* | |
| E.F. | 4.75 | 3.7 | 0.01 |
| M.H. | 3.6 | 2.0 | 0.003 |
| M.W. | 4.0 | 3.1 | 0.05 |
| M.F. | 3.6 | 2.9 | 0.05 |
| J. McF. | 3.1 | 2.1 | 0.03 |
| I.C. | 5.4 | 4.2 | 0.01 |
| A.P. | 4.6 | 3.6 | 0.002 |
| M.D. | 3.6 | 2.5 | 0.04 |
| J.C. | 4.3 | 3.4 | 0.02 |
| A.A. | 3.6 | 2.5 | 0.004 |
| P.G. | 4.0 | 2.4 | 0.00008 |
| E.A. | 5.0 | 4.2 | 0.01 |
| H.C. | 4.1 | 2.9 | 0.001 |
| J.A. | 4.1 | 0 | [a] |
| E.B. | 4.3 | 2.7 | 0.004 |
| W.S. | 3.5 | 3.0 | 0.03 |
| J.E. | 4.2 | 3.6 | 0.00008 |
| M.F. | 6.9 | 2.0 | 0.0007 |

[a]Denotes no $P$ value available due to too few degrees of freedom but tumour clinically impalpable at treatment week 12.

patients who progressed on treatment, except one, had tumours with an ER concentration of less than 20 fmol mg$^{-1}$ cytosol protein (Figure 3). This relationship held true for both premenopausal and post-menopausal patients.

*Relationship between response and disease-free survival*

With a mean follow-up period of 40.6 months, eight patients have developed recurrent disease; two patients who responded to endocrine therapy and six patients who did not. Clinical response is related to disease-free survival in Figure 3. Using the generalised Wilcoxon (Breslow) survival test, there was a statistical significant difference in survival between those patients who showed a response to endocrine treatment and those who did not ($P<0.05$).

**Table III** Response rates of 43 patients with large operable breast carcinoma treated with primary endocrine therapy

| | *Total no. of patients* | *ER < 20*[a] *(no. patients responding/ total)* | *ER ≥ 20*[a] *(no. patients responding/ total)* |
|---|---|---|---|
| Premenopausal | | | |
| Oophorectomy | 4 | 0/2 | 1/2 |
| GnRH analogue | 13 | 0/3 | 5/10 |
| Total | 17 | 0/5 | 6/12 |
| Post-menopausal | | | |
| Tamoxifen | 10 | 0/4 | 4/6 |
| Aminoglutethimide | 9 | 0/4 | 4/5 |
| 4-Hydroxyandrostenedione | 7 | – | 4/7 |
| Total | 26 | 0/8 | 12/18 |
| Grand total | 43 | 0/13 | 18/30 |

[a]fmol mg$^{-1}$ cytosol protein.

**Figure 2** The relationship between the pretreatment ER concentration in 43 patients with primary operable carcinoma of the breast and clinical response to 3 months' treatment with endocrine therapy. The ER concentration was determined by the dextran-coated charcoal adsorption method. Response was assessed from statistical analysis of the regression slope (Student's *t* test, see text). The median ER concentration in each response group is shown by a bar. Open circles have been used to denote tumours from premenopausal patients and closed circles tumours from post-menopausal patients.

**Figure 3** The relationship between disease-free survival and response to endocrine therapy in 43 patients with large but operable primary carcinoma of the breast. With a mean follow-up period of 40.6 months (range 7–43 months), there is a statistically significant difference in the survival of those patients who responded to treatment when compared to those who did not ($P<0.05$, generalised Wilcox (Breslow) survival test).

### Discussion

This report confirms and expands our previous reports (Forrest *et al.*, 1986; Hawkins *et al.*, 1988; Anderson *et al.*, 1989) of the relationship between ER concentration and response to endocrine therapy in primary operable breast cancer. Primary tumours with an ER concentration of less than 20 fmol mg$^{-1}$ cytosol protein did not respond to endocrine therapy, while 60% of those with higher ER values did. Using an immunocytochemical assay we have observed a similar relationship between ER status and response of primary tumours in elderly women treated with tamoxifen (Gaskell *et al.*, 1989). Considering that this relationship also exists for metastatic disease (McGuire *et al.*, 1975; Brooks *et al.*, 1980; Jensen, 1975; Dao & Nemoto, 1980; Oriana *et al.*, 1987), it is reasonable to expect that ER concentration should be of value in selecting appropriate adjuvant endocrine therapy. We have already discussed the confusion which surrounds the value of ER activity in predicting benefit with adjuvant tamoxifen. *In vitro* studies have suggested that tamoxifen may have antitumour actions which are independent of the oestrogen receptor (Sutherland *et al.*, 1986) and can be distinguished from nonspecific cytotoxicity by its cell cycle specific nature (Sutherland *et al.*, 1983). Thus results obtained using adjuvant tamoxifen cannot necessarily be extrapolated to other forms of endocrine therapy, e.g. oophorectomy. Further definition of the role of ER activity in predicting the benefit from adjuvant endocrine therapy is obviously required. This can only come from well conducted, controlled randomised trials designed specifically to answer this question and in which the performance of ER assays is of uniform high quality.

Systemic therapy is of major importance in the long term control of invasive breast cancer because of the high possibility of micrometastatic disease at the time of initial presentation (Brinkley & Haybittle, 1984). Since it is not yet possible to predict, on an individual basis, those patients who will benefit from a specific form of systemic therapy, there is theoretical value in giving systemic therapy as the preferred first line treatment. This would enable the response of the individual's tumour to be assessed and allow selection of appropriate systemic therapy. The availability of fine-needle aspiration techniques for diagnosis (Dixon *et al.*, 1984) and more recently ER assay (Coombes *et al.*, 1987; Hawkins *et al.*, 1988; Anderson *et al.*, 1989; Gaskell *et al.*, 1989) should lend feasibility to this approach, avoiding the need for open biopsy.

We thank Miss Ann Tesdale, Mr W. Ferguson and Mr D. Carson of the Department of Surgery who performed the ER assays; Drs T.J. Anderson and Dr J. Going, Department of Pathology, Dr A. Kirkpatrick, Department of Medical Radiology and all of the medical and nursing staff of Longmore hospital for their care and concern. This research was supported by a grant from the Cancer Research Campaign (SP1256). The study was approved by the hospital ethical committee.

### References

ANDERSON, E.D.C., HAWKINS, R.A. & FORREST, A.P.M. (1989). Oestrogen receptors and primary breast cancer: correlation of cytochemical with biochemical assay and subsequent response to hormonal therapy. *Br. J. Surg.* (in the press).

BIANCO, A.R., DE PLACIDO, S., GALLO, C. and 5 others (1988). Adjuvant therapy with tamoxifen in operable breast cancer. Ten year results of Naples (Gun) study. *Lancet*, **i**, 1095.

BRADFORD, M.M. (1976). A rapid and sensitive method for the quantitation of microgram quantities of protein utilising the principle of protein-dye binding. *Anal. Biochem.*, **72**, 248.

BRINKLEY, D. & HAYBITTLE, J.L. (1984). Longterm survival of women with breast cancer. *Lancet*, **i**, 1118.

BROOKS, S.C., SAUNDERS, D.E., SINGHAKOWINTA, A. & VAITKEVICIUS, V.K. (1980). Relation of tumor content of estrogen and progesterone receptors with response of patient to endocrine therapy. *Cancer*, **46**, 2775.

COOMBES, R.C., POWLES, T.J., BERGER, U. and 5 others (1987). Prediction of endocrine response in breast cancer by immunocytochemical detection of oestrogen receptor in fine-needle aspirates. *Lancet*, **ii**, 701.

DAO, T.L. & NEMOTO, T. (1980). Steroid receptors and response to endocrine ablations in women with metastatic cancer of the breast. *Cancer*, **46**, 2779.

DIXON, J.M., ANDERSON, T.J., LAMB, J., NIXON, S.J. & FORREST, A.P.M. (1984). Fine needle aspiration cytology, in relationships to clinical examination and mammography in the diagnosis of a solid breast mass. *Br. J. Surg.*, **71**, 593.

FISHER, B., REDMAN, C., BROWN, A. and 8 others (1986). Adjuvant chemotherapy with and without tamoxifen in the treatment of primary breast cancer: 5-year results from the National Surgical Adjuvant Breast and Bowel Projecy Trial. *J. Clin. Oncol.* **4**, 459.

FORREST, A.P.M., LEVACK, P.A., CHETTY, U. and 4 others (1986). A human tumour model. *Lancet*, **ii**, 840.

GASKELL, D.J., HAWKINS, R.A., SANGSTER, K. & FORREST, A.P.M. (1989). Immunocytochemical estimation of oestrogen receptors in elderly patients with primary breast cancer: relevance to treatment with tamoxifen. *Lancet* (in the press).

HAWKINS, R.A., BLACK, R., STEELE, R.J.C., DIXON, J.M.J. & FORREST, A.P.M. (1981). Oestrogen receptor concentration in primary breast cancer and axillary node metastases. *Breast Cancer Res. Treat.*, **1**, 245.

HAWKINS, R.A., SANGSTER, K., TESDALE, A.L. and 4 others (1987*a*). Experience with new assays for oestrogen receptors using monoclonal antibodies. *Biochem. Soc. Trans.*, **15**, 949.

HAWKINS, R.A., WHITE, G., BUNDRED, N.J. and 4 others (1987*b*). Prognostic significance of oestrogen and progestogen receptor activities in breast cancer. *Br. J. Surg.*, **74**, 1009.

HAWKINS, R.A., SANGSTER, K., TESDALE, A. and 4 others (1988). The cytochemical detection of oestrogen receptors in fine needle aspirates of breast cancer; correlation with biochemical assay and prediction of response to endocrine therapy. *Br. J. Cancer*, **58**, 77.

JENSEN, E.V. (1975). Estrogen receptors in hormone-dependent breast cancers. *Cancer Res.*, **35**, 3362.

MARSHALL, J.S., GORDON, N.H., HUBAY, C.A. & PEARSON, O.H. (1987). Assessment of tamoxifen as adjuvant therapy in stage II breast cancer; a long term follow-up. *J. Lab. Clin Med.*, 300.

MEAKIN, J.W. (1986). Review of Canadian trials of adjuvant endocrine therapy for breast cancer. *NCI Monographs*, **1**, 111.

McGUIRE, W.L., PEARSON, O.H. & SEGALOFF, A. (1975). Predicting hormone responsiveness in human breast cancer. In *Estrogen Receptors in Human Breast Cancer*, McGuire, W.L., Carbone, P.P. & Vollmer, E.P. (ed) p. 17. Raven Press: New York.

NOLVADEX ADJUVANT TRIAL ORGANISATION (1988). Controlled trial of tamoxifen as single adjuvant agent in management of early breast cancer. Analysis at eight years. *Br. J. Cancer*, **57**, 608.

ORIANA, S., SECRETO, G., DI FRONZO, G. & TORRI, A. (1987). Urinary androgens and tumour estrogen receptor as predictors of ovarian response and of survival in advanced breast cancer. *Breast Cancer Res. Treat.*, **9**, 201.

PALSHOF, T., CARSTENSEN, B., MOURIDSEN, H.T. & DOMBERNOWSKY, P. (1985). Adjuvant endocrine therapy in pre- and postmenopausal women with operable breast cancer. *Rev. Endocrine-related Cancer*, suppl. **17**, 43.

ROSE, C., THORPE, S.M., ANDERSEN, K.W. and 4 others (1985). Beneficial effect of adjuvant tamoxifen therapy in primary breast cancer patients with high oestrogen receptor values. *Lancet*, **i**, 16.

RUTQUIST, L.E., CEDERMARK, B, GLAS, U. and 7 others (1987). The Stockholm trial on adjuvant tamoxifen in early breast cancer. Correlation between estrogen receptor level and treatment effect. *Breast Cancer Res. Treat.*, **10**, 255.

SCATCHARD, G. (1949). The attractions of proteins for small molecules and ions. *Ann. NY Acad. Sci.*, **51**, 660.

SCOTTISH CANCER TRIALS OFFICE (MRC) EDINBURGH (1987). Adjuvant tamoxifen in the management of operable breast cancer: the Scottish Trial Report from the Breast Cancer Trials Committee. *Lancet*, **ii**, 171.

SUTHERLAND, R.L., GREEN, M.D., HALL, R.E., REDDEL, R.R. & TAYLOR, I.W. (1983). Tamoxifen induces accumulation of MCF-7 human mammary carcinoma cells in the Go/G1 phase of the cell cycle. *Eur. J. Cancer Clin. Oncol.*, **19**, 615.

SUTHERLAND, R.L., WATTS, C.K. & RUENITZ, P.C. (1986). Definition of two distinct mechanisms of action of antioestrogens on human breast cancer cell proliferation using hydroxytriphenylethylenes with high affinity for the oestrogen receptor. *Biochem. Biophys. Res. Commun.*, **140**, 523.